# Remission of Type 2 Diabetes Mellitus after Bariatric Surgery: Fact or Fiction?

**DOI:** 10.3390/ijerph16173171

**Published:** 2019-08-30

**Authors:** Dimitrios Tsilingiris, Chrysi Koliaki, Alexander Kokkinos

**Affiliations:** First Department of Propaedeutic Internal Medicine and Diabetes Center, Medical School, National and Kapodistrian University of Athens, Laiko General Hospital, Athens 11527, Greece

**Keywords:** type 2 diabetes, diabetes remission, bariatric surgery, metabolic surgery

## Abstract

Although type 2 diabetes mellitus (T2DM) has been traditionally viewed as an intractable chronic medical condition, accumulating evidence points towards the notion that a complete remission of T2DM is feasible following a choice of medical and/or surgical interventions. This has been paralleled by increasing interest in the establishment of a universal definition for T2DM remission which, under given circumstances, could be considered equivalent to a “cure”. The efficacy of bariatric surgery in particular for achieving glycemic control has highlighted surgery as a candidate curative intervention for T2DM. Herein, available evidence regarding available surgical modalities and the mechanisms that drive metabolic amelioration after bariatric surgery are reviewed. Furthermore, reports from observational and randomized studies with regard to T2DM remission are reviewed, along with concepts relevant to the variety of definitions used for T2DM remission and other potential sources of discrepancy in success rates among different studies.

## 1. Introduction

During the last decades, obesity has reached epidemic proportions in both the developing and the developed world [1]. As a direct consequence, the global burden of obesity-related comorbidities has been substantially increased. Overall and visceral adiposity are implicated in the pathogenesis of insulin resistance and type 2 diabetes mellitus (T2DM) [2] and hence, epidemiological trends for obesity are paralleled by a propensity towards a higher prevalence of T2DM [3,4]. Although the slope of the linear relationship between excess body weight and T2DM is not identical across all ethnic groups, the causal link between these two conditions is such that they are often viewed as a joint burden, composing the global epidemic of “diabesity” [5,6,7,8].

The medical management of T2DM typically consists of lifestyle modifications and specific glucose-lowering medications. The latter are targeted at maintaining glucose levels within an acceptable range, while the former aim to achieve weight loss through diet, increased physical activity and behavioral therapy, in order to beneficially modulate the underlying pathophysiology of T2DM [9]. Although most individuals benefit from these conservative approaches in the short term, a durable and clinically significant weight loss and its associated metabolic improvement is rarely, if ever, achieved [10]. Furthermore, despite major advances in recent years, diabetes pharmacotherapy is often oriented towards managing only hyperglycemia, which could be considered to be the “tip of the iceberg” among the numerous metabolic perturbations of the disease.

The remarkable effects of bariatric surgery regarding sustained weight loss and metabolic amelioration have gradually gathered attention and highlight the potential of surgery to serve as a therapeutic modality for T2DM. By addressing various complementary pathogenetic mechanisms, bariatric surgery appears to be promising with regard to the reversal of the metabolic abnormalities leading to overt T2DM. The philosophy behind bariatric surgical procedures has gradually shifted from weight loss per se to targeting additional cardiometabolic improvement, and indications have been broadened to include individuals with varying levels of adiposity and poorly controlled T2DM [11]. For the first time in the history of diabetes therapy, a therapeutic approach has emerged, which holds promise not only as an effective management tool but also as a potential cure.

In the present review, the concept of what defines a cure or remission of T2DM is critically discussed. Furthermore, the available evidence regarding the effects of bariatric surgery on T2DM remission and its related pathophysiological mechanisms are concisely reviewed, along with the major factors that may predict T2DM remission following surgery.

## 2. Overview of Interventions

**Figure 1:**
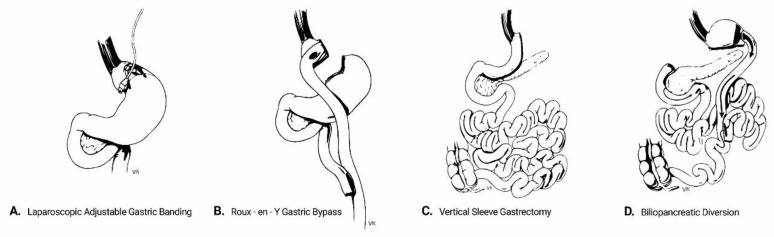
Schematic presentation of the main bariatric surgical modalities.

Although bariatric procedures were traditionally categorized into restrictive, malabsorptive or mixed, it has become clear that there are no definitive separating lines regarding the underlying mechanisms that drive weight loss and metabolic amelioration following different types of surgery [12]. Currently, the most commonly used bariatric procedures comprise the following (Figure 1):

- Laparoscopic adjustable gastric banding (AGB) refers to the placement of an adjustable silicon band around the upper part of the stomach. This isolates the gastric segment proximal to the band to create a small gastric pouch, thereby restricting the effective gastric volume. The size of the band and thus the degree of restriction can be adjusted by adding or removing saline solution through a subcutaneously inserted port. Despite being minimally invasive and deprived of major complications, AGB frequently fails to yield major results in magnitude and duration, owing to its one-dimensional approach and the potential for the patient to bypass the restriction by modifying diet quality towards more liquid foods. Its use is currently decreasing, being gradually displaced by the other bariatric procedures [13].
- Biliopancreatic diversion (BPD) is a mixed restrictive/malabsorptive procedure which was originally introduced by Scopinaro in 1979 [14]. It includes a partial horizontal gastrectomy and anastomosis of the gastric remnant in the distal 250 cm of the small intestine (alimentary limb), while the diverted proximal intestine carries biliopancreatic secretions. The latter is anastomosed to the alimentary limb at a varying distance from the ileocecal valve, which determines the degree of malabsorption. The procedure was later modified to include a vertical gastrectomy instead, with preservation of the pylorus and a duodeno-intestinal anastomosis, in order to prevent post-surgical dumping syndrome [15]. Although BPD has the highest success rates regarding weight loss and metabolic improvement among all bariatric procedures, its technical difficulties and high rates of perioperative and long-term complications confine its use to individuals with massive obesity or as salvage therapy after other procedures have failed.
- Roux-en-Y gastric bypass (RYGB) includes the creation of a small-volume gastric pouch which is anastomosed to the distal part of the jejunum (alimentary limb). The limb carrying biliopancreatic secretions is anastomosed typically 150 cm distally to the gastro-jejunostomy. RYGB has a balanced safety-efficacy profile and is considered to be the “gold standard” in the surgical therapy of obesity and T2DM [16].
- Vertical sleeve gastrectomy (VSG) involves the resection of the major proportion of the fundus and corpus of the stomach, leaving a tube-shaped gastric residue. It was originally the first part of a two-step approach for biliopancreatic diversion in high-risk individuals. Its effectiveness and operative simplicity have led to VSG being performed as a stand-alone procedure. It is currently the most common procedure among all bariatric modalities in the USA [13].

The most commonly performed bariatric operations comprise VSG and RYGB [13]. All types of surgery are currently performed laparoscopically, unless otherwise indicated.

## 3. Mechanisms of Type 2 Diabetes Mellitus (T2DM) Remission Following Bariatric Surgery

A cascade of anatomical and physiological alterations and subsequent metabolic adaptations exert beneficial effects on insulin production and sensitivity after bariatric surgery, leading to amelioration of hyperglycemia or even restoration of euglycemia. Although from a clinical point of view the distinction between the separate mechanisms of metabolic improvement following surgery is not always feasible or necessary, the major contributory factors can be separated into those related to weight loss and those that are weight-loss independent.

There is a continuous relationship between weight loss and the consequent improvement in glycemic levels and the probability of T2DM remission. The results of the The Diabetes Remission Clinical Trial (DiRECT), in which lifestyle measures consisting primarily of a stringent dietary management and increased physical activity were implemented, provide a striking example for this notion; in the DiRECT trial, there was an ascending probability of T2DM remission across categories of increasing absolute weight loss, ranging from 3.6% to 86.1% at the end of the first year and 5.2% to 70% at the end of the second year, for individuals losing < 5 kg and ≥ 15 kg of body weight, respectively [17]. Of note, there was a low absolute number of participants achieving a notable loss of weight in the cohort of DiRECT participants; just over 15% of participants retained a loss of ≥ 15 kg after 2 years while over half did not achieve relevant weight loss (< 5 kg) [18]. In the case of bariatric surgery, the major drive for weight loss is the post-operative establishment of a state of profound negative energy balance, leading to the long-term restoration of peripheral insulin sensitivity [19]. The sustained reductions in energy intake post-surgery primarily depend upon the reduction of hunger and induction of satiety. This may relate to either early post-prandial distention of a reduced-capacity upper gastrointestinal pouch sending satiety signals through the afferent vagal pathways, or to the modulation of hunger and satiety signaling networks in subcortical brain areas regulating energy intake. The latter mechanism involves an augmented post-prandial secretion of satiety-inducing gut peptides such as glucagon-like peptide 1 (GLP-1), peptide YY (PYY) and oxyntomodulin (OXM), combined with a diminished secretion of orexigenic hormones such as ghrelin and possibly altered leptin signaling in the hypothalamus [19]. Additional factors promoting weight loss after bariatric surgery include increased total energy expenditure and enhanced meal-induced thermogenesis [20,21,22], post-surgical changes in gut microbiota and altered bile acid physiology [19].

The fact that an improvement in glycemic indices is observed as early as within a few days post-operatively, before any clinically significant weight loss is achieved, suggests the presence of weight-loss independent mechanisms of metabolic amelioration following bariatric surgery. The acute drastic caloric restriction in the post-operative period may partly account for these effects, since it may contribute to normalization of plasma glucose levels, improvement in beta cell function, and enhancement of hepatic insulin sensitivity in patients with T2DM [23,24]. It could even restore the impaired first phase of insulin secretion, which is an early hallmark in the course of T2DM pathogenesis [23]. Gut peptide dynamics including humoral satiety signals and mediators of the incretin effect (which is impaired early in the course of T2DM) may also undergo robust changes following both VSG [25] and RYGB [26,27]. This is presumably due to the accelerated contact of ingested nutrients with specialized entero-endocrine cells residing in distal parts of the gastrointestinal tract (the so called “hindgut hypothesis”). Another putative mechanism regarding the observed metabolic reconstitution after bariatric surgery involves the anti-apoptotic effects of GLP-1 and PYY on pancreatic beta cells [28,29,30]. Although most available data are derived from in vitro and animal studies, the increased GLP-1 and PYY levels after surgery may contribute to the preservation of beta cell mass and function in the long term by inhibiting beta cell apoptosis [31,32,33]. Apart from adaptations in post-prandial gut peptide responses, additional humoral mechanisms may contribute to the restoration of insulin sensitivity following surgery. Perakakis et al. have recently shown that reduced circulating follistatin levels 3 months after RYGB or VSG are able to predict improvements in fasting plasma glucose (FPG), glycated hemoglobin (HbA1c) and insulin resistance 12 months post-operatively, both in individuals with and without T2DM [34]. Additional factors with a presumed weight-loss-independent role in metabolic improvement post-surgery include increased glucose utilization from the intestine, and alterations of gut microbiota [35].

## 4. The Challenge of Defining T2DM Remission

T2DM diagnosis is based on sharply defined cut-off values of laboratory parameters determined through standardized and quality-controlled methods [36]. In general, these criteria are widely accepted by national diabetes societies worldwide and implemented for use in the corresponding local guidelines. Moreover, the “gray zone” of glycemic indices also known as prediabetes, is also sharply delineated by glucose and HbA1c values belonging neither to the normoglycemic nor to the T2DM range. All this leaves little margin for debate over the identification of an individual’s position across the spectrum of glycemic control.

In contrast, there has been substantially less homogeneity with regard to the criteria defining diabetes remission, or even the potential to characterize the restoration of normoglycemia in an individual previously diagnosed with T2DM. In general, T2DM has been historically considered an intractable condition. Its overt manifestation marks a decline of beta cell function below a certain threshold, where hyperglycemia emerges as a consequence of both reduced glucose uptake from insulin-resistant peripheral tissues and increased hepatic glucose output. From this point on, even if hyperglycemia is adequately controlled by antidiabetic agents, there is further ongoing loss of beta cell mass and function [37]. Given that the vicious circle of insulin resistance, hyperinsulinemia and loss of beta cell function eventually leading to overt T2DM, may be tackled by addressing the factors that contribute to insulin resistance, there is a pathophysiological rationale for the interception of the pathogenesis of T2DM or even its reversal. This could be defined as a restoration of an energy homeostatic state in which the available secretory reserves of the pancreatic beta cells would suffice to maintain plasma glucose levels below the diabetic threshold, in the absence of the need for antidiabetic agents for a considerable period of time.

The most widely accepted definition for T2DM remission emerged from an American Diabetes Association (ADA) multidisciplinary expert panel consensus in June 2009 [38]. After addressing the challenges implicated in the delineation of a cure or remission of T2DM, this panel provided a definition based on three criteria: glycemia below diabetic range, absence of treatment, and sustainability over time. Specifically, provided that there is no ongoing drug treatment, diabetes remission was defined as:

- Partial, when glycemic indices fall into the pre-diabetic range (HbA1c 5.7–6.4%, FPG 100–125 mg/dL) for at least 1 year;
- Complete, when normoglycemia is restored (HbA1c < 5.7%, FPG < 100 mg/dL) for at least 1 year;
- Prolonged, defined as a complete remission of at least 5 years’ duration.

These definitions do not distinguish between the two major types of diabetes or the means by which remission is achieved. The panel has also provided recommendations regarding the need for continued screening for diabetes-related complications [38]. Of note, no consensus was reached regarding glucose values obtained during an oral glucose tolerance test (OGTT). Still, the straightforwardness and ease of application of these criteria allow for their broad-scale use for “screening” and monitoring changes during long-term follow up, as well as for assessment of the effects of different treatments on diabetes remission. Nevertheless, they are subject to certain drawbacks similar to those acknowledged for their use in diabetes screening, i.e., pre-analytical instability for FPG [39], and biological and analytical variability for HbA1c [40]. In the case of bariatric surgery, additional caution is warranted when using HbA1c as an index of remission, since certain clinical conditions with the potential to affect HbA1c values (and consequently compromise its utility as an indicator of glycemic levels) may occur more frequently in bariatric surgery patients (i.e., iron or vitamin B12 deficiency) [40,41]. Another area of potential controversy that most T2DM remission definition approaches share is the lack of a specified minimum timeframe that would be considered essential for diabetes medication to have been discontinued (“off-treatment” status).

Among studies reporting T2DM remission rates after medical or surgical interventions, there is a substantial lack of agreement regarding the criteria used to define remission. This is also the case for studies published after the establishment of the ADA consensus criteria (Table 1).

## 5. Observational Studies and Randomized Clinical Trials (RCTs) Regarding T2DM Remission

The earliest documentation of the “curative” potential of the RYGB procedure for T2DM and other obesity-related comorbidities was published even before the results of the United Kingdom Prospective Diabetes Study (UKPDS) irreversibly changed the landscape of T2DM therapeutics. The breakthrough study by Pories et al. reported the results of a long-term follow up of 608 morbidly obese patients post-RYGB, 27% of whom had T2DM and another 27% impaired glucose tolerance (IGT). The authors found that by the end of the 14-year follow up, 82.9% of T2DM and 98.7% of IGT patients maintained FPG and HbA1c values within the euglycemic range [42]. There is no doubt that the definition of euglycemia and even more so the quality control of HbA1c laboratory measurements were not as standardized as nowadays. However, this study acted as a paradigm shift for the years to follow. The authors also recognized older age and longer T2DM duration as factors predicting the persistence of hyperglycemia [42].

The ongoing prospective, multicenter, non-randomized Swedish Obese Subjects Study (SOS), followed 2010 surgically treated obese patients (RYGB, AGB or vertical banded gastroplasty (VBG)) and 2037 conventionally managed matched controls over a period of over two decades [68]. Among the outcomes of the study was the incidence of new cases of T2DM, T2DM remission and relapse and incidence of diabetes complications. Regarding the potential of T2DM prevention through bariatric surgery, over a median follow up of 10 years, the incidence rate of new cases of T2DM was 6.8 cases per 1000 person-years and 28.4 cases per 1000 person-years after surgery and conservative management, respectively [hazard ratio (HR) 0.17 (95% confidence interval (CI) 0.13 to 0.21)] [53]. In the same cohort, the effect of surgery on diabetes remission and complications prevention was assessed in a subpopulation of individuals with T2DM at baseline (343 in the surgery arm and 260 in the control group). Diabetes remission rates at 2, 10 and 15 years of follow up were 72.3%, 38.1% and 30.4% respectively, significantly higher than those observed in the conventionally treated arm. Although the surgically treated arm consisted of individuals having undergone a variety of operational modalities (RYGB, AGB or VBG), there was no impact of type of surgery on T2DM remission. It should be also noted that the criterion for T2DM remission was FPG < 110 mg/dL in the absence of antidiabetic treatment [69]. After a median follow up of 18.1 and 17.6 years for surgically and conventionally treated patients respectively, the rate of diabetes complications was significantly lower in the surgical arm (cumulative incidence 20.6 vs. 41.8 per 1000 person-years for microvascular and 31.7 vs. 44.2 for macrovascular complications) [69].

In a prospective Utah-based study, Adams et al. examined the effects of RYGB among 1146 individuals with at least grade II obesity (418 RYGB, 417 controls seeking but not undergoing surgery, 321 controls selected from a population-based sample, 88, 93 and 88 with T2DM at baseline, respectively). At the end of the 6-year follow up, participants in the RYGB arm had lost an average of 27.7% of their initial body weight compared with a gain of 0.2% and 0% for the two control groups. Using the “normalization” of FPG and HbA1c values in the absence of antidiabetic treatment as a diabetes remission criterion, the authors reported T2DM remission rates of 75% and 62% at 2- and 6-years post-surgery for the surgically treated arm, significantly higher than those observed in the control groups. A significantly lower incidence of new cases of T2DM was also found in the surgical group [54]. A previous retrospective study of the same group examined the effect of RYGB on various long-term outcomes in a cohort of 7925 patients undergoing RYGB and an equal number of age-, gender- and body mass index (BMI)-matched morbidly obese untreated control subjects. After a median follow up of 7.1 years, the authors reported a 92% reduction in diabetes-related deaths in operated patients [70].

In a non-randomized controlled study, Iaconelli et al. aimed to examine the effect of BPD versus conventional antidiabetic management on the incidence of long-term diabetic complications among 110 newly diagnosed T2DM individuals with at least grade II obesity. T2DM remission was a secondary endpoint of this study. At the end of the first year post-surgery, all BPD patients had experienced diabetes remission, which was further maintained throughout the 10-year follow up for all patients who completed the study, fulfilling the criterion of a prolonged remission as defined by the ADA [38]. The corresponding rate in the conventionally treated group was 45%. At the end of the follow up period, remission rates of T2DM and weight loss were significantly higher in the BPD group. These effects were accompanied by a regression of microalbuminuria and a lower incidence and progression of nephropathy, as well as by a lower incidence of coronary artery disease [49].

The Surgical Treatment and Medications Potentially Eradicate Diabetes Efficiently (STAMPEDE) trial is a study designed to examine the efficacy of bariatric surgery plus medical management compared to optimal medical management alone for glycemic control among poorly controlled T2DM individuals. Features that designate STAMPEDE as a landmark study in the field include its randomized controlled design (participants randomized in the RYGB, VSG and control arms in a 1:1:1 ratio), the inclusion exclusively of participants with T2DM, the ongoing long-term follow up of the cohort, the utilization of the most commonly used bariatric surgical modalities (VSG and RYGB) [13], and most importantly, its primary endpoint which is the percentage of participants with HbA1c < 6% irrespectively of active antidiabetic treatment. Although this endpoint does not fall into the ADA consensus definition for remission, it can still be viewed as a composite of the achievement of optimal glycemic control post-surgery. In other words, STAMPEDE is a RCT testing the hypothesis that bariatric surgery may have a “curative” potential for T2DM. Across RYBG, VSG and control groups, the primary endpoint occurred in 42%, 37% and 12% at the end of the first year, 28%, 24% and 5% at the end of the third year, and 29%, 23% and 5% at the end of the fifth year post-surgery, with the differences being statistically significant for both surgically treated groups vs. controls [51,71,72]. The use of glucose-lowering medications was lower among individuals in the RYGB and SG groups compared to controls at all studied time points [51,71,72]. Regarding the outcome of glycemic control without the use of antidiabetic agents, which corresponds to HbA1c values consistent with complete T2DM remission according to the ADA definition, the corresponding rates were 42%, 27% and 0% at year 1, 35%, 20% and 0% at year 3 and 22.4%, 14.9% and 0% at year 5, respectively [51,71,72]. The percentages of patients with HbA1c < 6.5% without the use of antidiabetic agents, equivalent to HbA1c values for partial or complete remission, were 46%, 29% and 0% at year 3, and 30.6%, 23.4% and 0% at year 5, respectively [71,72]. 

A preceding RCT by Dixon et al. compared the efficacy of AGB versus conventional medical treatment for T2DM remission (defined as FPG and HbA1c values < 126 mg/L duration < 2 years). After 2 years of follow up, remission rates were 73% in the AGB group and only 13% in the medical management group. Of note, the probability of remission was positively related to the magnitude of weight loss and negatively related to baseline HbA1c [48].

Another landmark study in the field by Mingrone et al. compared T2DM remission rates (FPG < 100 mg/dL and HbA1c < 6.5% without antidiabetic treatment for at least 1 year) between three groups of patients (RYGB, BPD and conservatively treated) with T2DM of at least 5 years’ duration, preoperative BMI of ≥ 35 kg/m^2^ and HbA1c values ≥ 7% [52]. The reported remission rates were 95%, 75% and 0% at 2 years for the BPD, RYGB and conservative arms, respectively [52], which was sustained in 63% and 39% of patients after 5 years for BPD and RYGB, respectively [73]. Despite the observed rate of relapse, overall glycemic control with or without treatment and medication use were significantly lower in the surgically treated arms, while diabetes-related complications were observed only in the conservatively treated group [73].

The CROSSROADS trial (Calorie Reduction Or Surgery: Seeking to Reduce Obesity And Diabetes Study) compared the effects of RYGB versus an intensive non-surgical medical therapy combined with lifestyle intervention on T2DM remission (defined as HbA1c < 6% off antidiabetic medication), among individuals with T2DM and a baseline BMI ranging between 30 and 45 kg/m^2^. T2DM remission rates were 60% and 5.9% for the RYGB and non-surgical arms, respectively. Of note, individuals in the RYGB-treated group had longer T2DM duration, higher baseline HbA1c and greater use of insulin than the control group [64].

The superior efficacy of bariatric surgery to induce diabetes remission compared to conservative management has been further demonstrated in subjects within the overweight range. A RCT by Wentworth et al. examined the metabolic effects of AGB when added to multidisciplinary diabetes care in overweight individuals with T2DM. At two years, T2DM remission rates were 52% in the surgical versus 8% in the control group receiving conservative treatment only. Remission was defined as glucose values in the non-diabetic range during an OGTT performed at least two days after discontinuation of glucose-lowering medications [59].

Another RCT by Courcoulas et al. compared the effects of RYGB, AGB and non-surgical treatment on T2DM remission (as defined by the ADA) among individuals with T2DM and obesity grades I-II. The rates of partial and complete T2DM remission after 1 year of follow up were 50/17%, 27/25% and 0/0% for the RYGB, AGB and medically treated arms, respectively [60]. After 3 years of follow up, remission (partial and complete) within the cohort was 40%, 29% and 0% for RYGB, AGB and the control group, respectively [74].

## 6. Factors that Predict T2DM Remission after Bariatric Surgery

Not all individuals with T2DM experience remission after bariatric surgery. Unsurprisingly, the improvement of glycemic control relates to the degree of weight loss after surgery [75], while less profound weight loss during the first postoperative year and greater weight regain may predict T2DM relapse [76].

When considering surgery for a patient with T2DM as a potentially “curative” option, it would be of major importance to determine potential preoperative characteristics at patient level which could reliably predict diabetes remission following surgery. Table 2 reviews patient-level characteristics identified as predictive of T2DM remission following surgery among different studies.

As early as in the pioneer study by Pories et al., non-responders to bariatric surgery were identified to be of older age and longer T2DM duration than those who were restored to euglycemia. It was unclear, however, whether the effects of age and T2DM duration were independent [42]. Accumulating evidence from landmark studies in the field consolidated early observations and contributed to the development of prognostic scoring systems. In a retrospective analysis of 505 obese T2DM patients undergoing RYGB, Blackstone et al. reported a complete remission rate of 43.2% 14 months after surgery, while a longer T2DM duration, insulin use and poor preoperative glycemic control were identified as predictors of non-remission [77]. Chikunguwo et al. analyzed data from 177 patients with T2DM who underwent RYGB and for whom long-term (> 5 years) follow up data were available. T2DM remission was defined as the lack of need for antidiabetic treatment at any time point during the postoperative course, while durable remission was defined as the absence of signs of T2DM without ongoing treatment for >5 years. Durable remission and lack of recurrence were observed more commonly among patients of younger age and those treated with diet or oral antidiabetic agents only, while weight regain was a significant, albeit weak, predictor of T2DM recurrence [78].

A number of scoring systems have been developed in an attempt to integrate patient-related factors associated with the probability of T2DM remission into prognostic models [79]. Using data from a retrospective cohort study of RYGB-treated obese patients with T2DM, Still et al. identified four preoperative clinical variables (insulin treatment, patient age, HbA1c and type of antidiabetic agents), which were integrated into a scoring system (DiaRem score) to calculate the probability of T2DM remission (partial or complete as defined by the ADA) 5 years after surgery. The calculation of DiaRem score stratifies individuals within 5 categories of ascending score, providing a probability of remission achievement [77]. The DiaRem score is a practical clinical tool and has undergone external validation [80,81,82]. Although its application cannot be directly extrapolated to types of surgery other than RYGB, there is limited data supporting its applicability to VSG and possibly also to AGB [83,84]. Aron-Wisnewsky et al. added two more factors to DiaRem (duration of T2DM and number of glucose-lowering agents) and generated the Ad-DiaRem score, which has improved prognostic accuracy especially among individuals in the mid-range of DiaRem [85,86].

Notably, most available evidence suggests that baseline preoperative BMI is not predictive of glycemic improvement after surgery [87,88], although a minority of reports suggest a more modest effect on T2DM remission for individuals in the overweight or obesity grade I range, compared with their morbidly obese counterparts [89,90]. This observation has potential implications regarding the current BMI-centered criteria of eligibility for bariatric surgery [11]. To date however, this matter remains largely unresolved.

Regarding the effects of different types of surgery on the probability of T2DM remission, it is widely accepted that the efficacy of surgical modalities in order of descending magnitude is BPD, RYGB, VSG and finally AGB, which parallels the magnitude of excess weight loss expected from each procedure [63,88,91,92]. In addition, the different effects between RYGB and VSG in achieving T2DM remission may be partly accounted for by differential effects on specific aspects of T2DM pathophysiology, such as postprandial dysmetabolism. Of note, RYGB has shown superior efficacy compared to VSG in improving postprandial hyperglycemia and hypertriglyceridemia in morbidly obese patients, at least in the short term [93]. The relative contribution of weight loss and other weight loss-independent mechanisms to achievement of T2DM remission after each type of surgery is not always possible to quantify.

## 7. Sources of Heterogeneity in Reported T2DM Remission Rates among Studies

The reported results regarding the success rate in inducing T2DM remission vary significantly among different studies, even among those performing the same surgical procedure. There are multiple sources of heterogeneity contributing to these discrepancies. The most obvious relates to the definition used to describe T2DM remission. In general, the remission endpoints of most studies include the restoration of glycemia to non-diabetic levels in the absence of active pharmacotherapy, but even this is not universally the case, as in the STAMPEDE trial [51]. The cut-off values for FPG and HbA1c used in different studies also vary considerably (Table 1). The most stringent criteria are those introduced by the ADA consensus panel in 2009 [38]. Pournaras et al. conducted a retrospective analysis of data from 209 patients with T2DM undergoing bariatric surgery and reported lower T2DM remission rates using the ADA definition of complete remission compared with the previously applied criteria (HbA1c < 6% or FPG < 100 mg/dL). In the case of RYGB, this difference was found to be statistically significant [94]. A small prospective study by Mas-Lorenzo et al. yielded similar results [95]. Based on the above, the uniform application of the ADA remission criteria in future studies may attenuate this source of bias.

The different duration of follow up may also affect the reported rates of T2DM remission in different studies. The peak weight loss effects of bariatric surgery are typically observed between 12 and 18 months following surgery [96,97]. Since several individuals regain a proportion of their lost body weight in the long term, a factor which may lead to T2DM relapse, differences in the time points of postoperative evaluation between studies are bound to have an effect on the reported results. 

When T2DM remission rates after surgery are reported in comparison to a control intervention, the exact nature of the non-surgical treatment (lifestyle and/or medical T2DM management), its intensity (intensive management vs. routine care) or even the absence of a comparator intervention (i.e., age-, gender- and BMI- matched untreated control subjects) should be all taken into consideration [48,51,52,68,70]. It should be also considered whether the same measures have been also applied in the surgical arm additionally to surgery [51,59].

Furthermore, differences in study population characteristics may account for some degree of variability in the reported results. This is particularly true for the features known to affect T2DM remission rates after surgery; differences regarding participants’ T2DM duration, age, preoperative glycemic control and the type of treatment may have a significant impact on the observed outcomes. Finally, the sample composition regarding the relative representation of different ethnic groups may also affect the observed effects of surgery on T2DM remission [98,99].

## 8. Effect of Bariatric Surgery on Chronic T2DM Complications, Mortality and Quality of Life

Available data support the notion that bariatric surgery is associated with lower overall mortality and a lower incidence of both microvascular and macrovascular complications of T2DM [49,67]. A retrospective study by Coleman et al. that included data on 4683 obese individuals with BMI > 35 kg/m^2^ and T2DM who underwent bariatric surgery (RYGB, VSG or AGB) concluded that those who experience T2DM remission after either type of surgery have a 29% lower probability of developing diabetic microvascular disease compared to refractory cases [100]. Moreover, the rate of microvascular complications was lower even among those in which T2DM recurred after an initial remission, with a 19% reduction of complication rate for each year spent in remission, suggesting the presence of a legacy effect of bariatric surgery regarding microvascular complications [100]. However, most available evidence is derived from observational studies, and RCTs designed to address the effects of surgery on diabetes complications are currently lacking. Table 3 reviews the evidence regarding the effect of bariatric surgery on diabetes-related complications derived from observational and randomized-controlled studies of bariatric surgery.

In the retrospective cohort study by Adams et al. among severely obese individuals, RYGB was found to reduce all-cause mortality by 40% compared with age-, sex-, and BMI-matched controls, after a median follow-up of 7.1 years. A substantial benefit was noted regarding mortality due to diabetes, with a reduction of 92% in diabetes-related deaths [70]. In a recent meta-analysis of studies conducted among individuals with T2DM, bariatric surgery was associated with significantly reduced mortality (odds ratio (OR) 0.34) and rate of macrovascular complications (OR 0.38) in comparison to medical treatment [101]. It is not clear however to what extent these benefits can be attributed to better glycemic control (including remission of T2DM) rather than to weight loss per se and/or improvements in other cardiometabolic risk factors following surgery. In a Swedish retrospective study that included 6132 obese subjects with T2DM who underwent RYGB and an equal number of medically managed controls matched on age, sex and BMI, the authors noted 49%, 58% and 59% relative risk reductions for non-fatal myocardial infraction, all-cause and cardiovascular mortality [102]. Subsequent causal mediation analysis within the same cohort, however, showed that these effects are mediated through sustained weight loss rather than return to amelioration of other known risk factors, including hyperglycemia [103]. Given that the causal nature of the relationship of hyperglycemia to macrovascular complications and mortality is not nearly as straightforward as that regarding microvascular disease, the relative contribution of the return to euglycemia to these findings is often impossible to quantify.

Likewise, regarding quality of life (QOL), there is an overall consensus for a beneficial effect of bariatric surgery on various measurable QOL aspects, although it is not obvious whether this is a result of restoration of euglycemia or weight loss. In the SOS cohort, the observed longitudinal trends regarding all QOL domains except anxiety following surgery were found to correlate with changes in body weight over time [104]. Five-year outcomes in the study by Mingrone et al. ascertained statistically significant improvement in all physical and emotional aspects assessed in both surgical arms compared to controls [73], whereas the reported beneficial effects during the five-year follow up in the STAMPEDE trial were somewhat more modest and focused on components of physical health [72]. Table 3 includes an overview of the chief studies evaluating the impact of bariatric surgery on quality of life in patients with T2DM.

## 9. Short- and Long-Term Risks

Early (30-day) perioperative mortality for bariatric surgery is estimated in the range of 0.1–1.1%, with higher rates for BPD and open vs. laparoscopic procedures [106,107,108] with a likewise lower rate for laparoscopic modalities regarding early surgical-related morbidity (such as pulmonary embolism, respiratory failure, renal events, sepsis, acute coronary syndromes, gastrointestinal bleeding, surgical wound infection or dehiscence) [106,109]. Long-term complications include internal and incisional hernias, intra-abdominal adhesions, anastomotic strictures and ulcers, kidney and biliary lithiasis, nutritional deficiencies and psychiatric complications [109]. Long-term malnutrition issues are of particular importance, and manifest as a result of reduced nutrient intake or excessive vomiting and/or impaired absorption. Due to the risk of complications related to micronutrient deficiency, such as anemia, peripheral neuropathy or loss of bone mass, nutritionist follow up is of crucial importance following surgery and chronic, often lifetime supplementation of nutritional supplements is warranted following procedures with a significant malabsorptive component [110]. Psychological well-being following surgery is a complex issue. A recent nation-wide study in Sweden reported a 33% increase of any psychiatric diagnosis among RYGB patients compared to age-, sex- and BMI-matched controls [111]. Certain psychiatric components, however, such as depressive symptoms, may be beneficially affected by surgery [112]. Individuals undergoing RYGB are at high long-term risk of alcohol and substance abuse [111,113,114]. Self-harm and suicide rates in this population are significantly higher in this population compared to the general population [113,115]. A recent meta-analysis reported a pooled prevalence of suicide of 0.3% following bariatric surgery [116]. Additionally, suicide rates were found to be higher among surgically treated individuals compared to medically managed controls in the SOS cohort and the Scandinavian Obesity Surgery Registry cohort, with the observed risks being independent of the outcome on weight loss [117]. Although the absolute risk of death from suicide is substantially low compared to the overall survival gains following surgery [70], a careful preoperative psychiatric evaluation and post-operative follow-up is warranted, especially for individuals with a background of psychiatric spectrum disorders and/or a positive history of self-harm behavior. 

Advanced age should not by itself preclude individuals from receiving surgical T2DM treatment, since early perioperative mortality and morbidity does not seem to be substantially affected by a patient age of 40 years and above [118]. Older patients (> 60 years) seem to benefit from clinically significant weight loss and improvements in obesity-related comorbidities [119,120,121], but should be subjected to thorough preoperative evaluation and overall risk assessment, similarly to younger age groups.

## 10. Remission of T2DM after Bariatric Surgery: Fact or Fiction?

The constantly increasing understanding of the complex pathophysiologic cascade leading to the development of T2DM, paralleled by advances in medical treatment and accumulating expertise in the field of bariatric surgery, have gradually resulted in a shift in the view of T2DM from a “treatable” towards a “potentially curable” chronic condition. Of crucial importance and particularly challenging is to establish what defines a “cure” of T2DM and to which extent this term can be used interchangeably with a prolonged restoration of euglycemia or T2DM “remission”, which is undeniably more feasible to objectify.

This is not merely a matter of reproducible and standardized reporting of study results. The statement that a chronic medical condition is cured may exert profound effects on the person that is “cured” in multiple aspects, namely psychosocial, financial, and most importantly, medical. In the case of T2DM, this is extremely significant; on the one hand, a “cure” does not confer “immunity” and a future relapse is always a possibility. On the other hand, T2DM should be viewed as an important risk factor rather than only a disease, since it represents a health condition from which serious and often life-threatening complications may originate, often many years after its onset.

Should a sustained remission be considered essentially equivalent to cure, T2DM may be considered a curable condition. This can also be achieved through intensive lifestyle management, as demonstrated in the Look AHEAD and DiRECT trials [18,55], or more rarely by routine medical care in community settings [122]. However, an overwhelming body of evidence from observational and randomized studies has shown that T2DM remission is achieved in a substantially greater frequency and predictability through bariatric surgery compared to any tested non-surgical comparator. In other words, while bariatric surgery is not the sole existing therapy for T2DM, it is by far the most effective available “curative” option for T2DM. Alongside these unequivocal clinical benefits, the most widely used surgical modalities aiming at T2DM control come with small, albeit existent risk of short and long-term adverse effects. The demonstration of a dramatic decrease in diabetes-related mortality following bariatric surgery [70] and the documented favorable effects exerted on the probability of chronic diabetic complications leave little room for doubt over the risk-to-benefit ratio profile [123] of bariatric surgery for T2DM treatment. Furthermore, surgical approaches are likely to be cost-effective strategies in the long term [124,125,126,127,128]. 

A tailored therapeutic scheme for T2DM should aim to minimize the long-term complications and improve the quality of life of affected individuals. The available evidence suggests that this can be achieved after bariatric surgery, in parallel with substantial weight loss and improvement of glycemic control. Available evidence points towards the idea that, should T2DM remission be achieved following surgery, there is a substantial decrease in the rate of chronic diabetic complications irrespective of a potential future recurrence [100]. However, even when complete remission (by whichever definition) is not achieved, clinically significant improvements in glycemic control after bariatric surgery are expected to confer a lower risk of chronic complications and reduced medication needs [73,77].

Currently, the potential benefits of a wide-scale integration of bariatric surgery in standard diabetes care is hindered by the poor penetration that surgical therapeutic options share in T2DM management. Even though there is still a lack of data derived from populations of individuals with T2DM, estimates in the United States show that just over 1% of a total of approximately 18 million individuals with class III obesity currently undergo bariatric surgery [13]. The identification of preoperative patient-level characteristics that signify the cases with the highest probability of being refractory may divert selected individuals towards more aggressive and effective surgical procedures, and hence improve the overall risk-to-benefit efficacy of the surgical approach to T2DM treatment.

In summary, although the claim that bariatric surgery is a definitive cure for diabetes would be an overstatement, the fact that it is to date by far the best “curative” tool for T2DM is far from fiction. Novel and upcoming medications, targeting the mechanisms through which bariatric surgery can lead to weight loss and metabolic amelioration (i.e., gut-peptide receptor single or multiple agonists), may constitute a notable competitor for surgery in the near future. Besides, the first positive results regarding the applicability of integrated surgical and gut-peptide mimetic medical therapeutic approaches for the most refractory or relapsed cases of T2DM have already emerged in the medical literature [129].

## Figures and Tables

**Table 1:** Major studies reporting type 2 diabetes mellitus (T2DM) remission rates after surgical or lifestyle interventions and the respective remission criteria.

Study	Study Population Characteristics	Study Design	Intervention	T2DM Remission Endpoint
Pories et al. (1995) [42]	morbidly obese, T2DM, prediabetes	Retrospective cohort	RYGB	“Normal” levels of FPG, HbA1c
Wittgrove et al. (2000) [43]	Morbidly obese	Prospective cohort	RYGB	Medication withdrawal and “normal” HbA1c
Dixon et al. (2002) [44]	BMI > 35 kg/m^2^, T2DM	Prospective cohort	AGB	“Normal” levels of FPG, HbA1c, fasting insulin, c-peptide
Sugerman et al. (2003) [45]	Morbidly obese	Retrospective cohort	GBP	FPG ≤ 120 mg/dL off medication
Schauer et al. (2003) [46]	morbidly obese, T2DM	Prospective cohort	RYGB	“Normal” levels of FPG, HbA1c, medication withdrawal
Scopinaro et al. (2005) [47]	obese, T2DM	Retrospective cohort	BPD	FPG < 110 mg/dL, ≥ 125 mg/dL for relapse
Dixon et al. (2008) [48]	BMI 30–40 kg/m^2^ T2DM duration < 2 years	RCT	AGB	FPG < 126 mg/dL, HbA1c < 6.2% off medication
Studies after ADA consensus panel definition (2009)				
Iaconelli et al. (2011) [49]	BMI > 35 kg/m^2^ newly diagnosed T2DM	Open case-control	BPD	ADA definition #
Kehagias et al. (2011) [50]	BMI < 50 kg/m^2^	RCT	VSG, RYGB	Glucose values below diabetic range during 2h-OGTT, off medication
Schauer et al. (2012) (STAMPEDE) [51]	BMI ≥ 30 kg/m^2^ uncontrolled T2DM	RCT	VSG, RYGB	HbA1c < 6%
Mingrone et al. (2012) [52]	BMI ≥ 5 kg/m^2^ T2DM duration ≥ 5 years, HbA1c ≥7%	RCT	BPD, RYGB	FPG < 100 mg/dL and HbA1c < 6.5% off medication for ≥ 1 year
Carlsson et al. (2012) (SOS cohort) [53]	BMI ≥ 30 kg/m^2^	Prospective cohort	RYGB, AGB or VBG	FPG < 110 mg/dL off medication
Adams et al. (2012) [54]	BMI ≥ 35 kg/m^2^	Prospective cohort	RYGB	“Normal” levels of FPG, HbA1c off medication
Gregg et al. (2012) (Look AHEAD) [55]	BMI ≥ 25 kg/m^2^	RCT	ILI	FPG < 126 mg/dL and HbA1c < 6.5% off medication
Arteburn et al. (2013) [56]	T2DM	Retrospective cohort	RYGB	ADA definition #
Liang et al. (2013) [57]	BMI ≥ 25 kg/m^2^ T2DM duration 5–10 years	RCT	RYGB, exenatide	Normal FPG, HbA1c off medication
Arteburn et al. (2013) [58]	BMI ≥ 35 kg/m^2^ T2DM	Retrospective cohort	RYGB, AGB, VSG, other	FPG < 126 mg/dL and/or HbA1c<6.5% off medication for ≥90 days
Wentworth et al. (2014) [59]	BMI 25–30 kg/m^2^	RCT	AGB	Glucose values below diabetic range during 2h-OGTT, 2 days off medication
Courcoulas et al. (2014) [60]	BMI 30–40 kg/m^2^	RCT	AGB, RYGB	ADA definition #
Halperin et al. (2014) [61]	BMI 30–42 kg/m^2^ T2DM duration ≥ 1 year	RCT	RYGB	FPG < 126mg/dL and HbA1c < 6.5%
Risstad et al. (2015) [62]	BMI 50–60 kg/m^2^	RCT	RYGB, BPD	ADA definition #
Yska et al. (2015) [63]	BMI ≥ 35 kg/m^2^	Retrospective cohort	RYGB, VSG, AGB, other	HbA1c < 6% off medication
Cummings et al. (2016) (CROSSROADS) [64]	BMI 30–45 kg/m^2^	RCT	RYGB	HbA1c < 6% off medication
Purnell et al. (2016) (LABS-2) [65]	BMI ≥ 30 kg/m^2^	Prospective cohort	RYGB, AGB	HbA1c < 6.5% or FPG ≤ 6.9 mmol/L off medication
Salminen, et al. (2018) (SLEEVEPASS) [66]	Morbidly obese	RCT	VSG, RYGB	ADA definition #
Lean et al. (2018) (DiRECT) [17]	BMI ≥ 30 kg/m^2^	RCT	ILI	HbA1c < 6.5%, at least 2 months off medication
Madesin et al. (2019) [67]	BMI ≥ 35 kg/m^2^	Population-based cohort	RYGB	HbA1c < 6.5% off medication or HbA1c < 6% on metformin monotherapy

ADA: American Diabetes Association; AGB: adjustable gastric banding; BMI: body mass index; BPD: biliopancreatic diversion; FPG: fasting plasma glucose; GBP: gastric bypass; ILI: intensive lifestyle intervention; OGTT: oral glucose tolerance test; RCT: randomized clinical trial; RYGB: Roux-en-Y gastric bypass; SG: sleeve gastrectomy; SOS: Swedish Obese Subjects; T2DM: type 2 diabetes mellitus; VBG: vertical banded gastroplasty. # See Section 4 for definition.

**Table 2:** Pre-operative, patient-level factors that predict diabetes remission following bariatric surgery, identified among different studies.

Study	Factors Predicting Remission	Factors Exerting a Neutral Effect
Pories et al. (1995) [42]	Shorter T2DM duration Younger age	
Dixon et al. (2002) [44]	Shorter diabetes duration	
Schauer et al. (2003) [46]	Better pre-operative glycemic control Absence of insulin treatment Shorter diabetes duration	
Dixon et al. (2008) [48]	Better pre-operative glycemic control	Sex, age, baseline BMI, baseline C-peptide level, time spent engaged in planned physical activity
Schauer et al. (2012) (STAMPEDE) [51]	Shorter T2DM duration	Age, sex, insulin use pre-op, baseline BMI, HbA1c, C-peptide, CRP, BP, lipids
Mingrone et al. (2012) [52]	Baseline triglyceride concentration	
Carlsson et al. (2012) (SOS cohort) [53]	Shorter T2DM duration, use of oral antidiabetic agents vs. no use, lower baseline glucose	Age, sex, baseline BMI, baseline insulin treatment
Gregg et al. (2012) (Look AHEAD) [55]	Shorter diabetes duration Better pre-operative glycemic control Absence of insulin treatment	Age, sex, race, baseline BMI, antihypertensive treatment, history of cardiovascular disease (CVD)
Arteburn et al. (2013) [56]	Male sex Shorter T2DM duration Better pre-operative glycemic control No use of oral antidiabetic agents or insulin treatment on baseline	Age
Arteburn et al. (2013) [58]	Younger age Shorter T2DM duration No use of oral antidiabetic agents or insulin treatment on baseline Better pre-operative glycemic control Higher baseline BMI	Sex
Cummings et al. (2016) (CROSSROADS) [64]		Age, sex, baseline BMI, diabetes duration, insulin usage
Purnell et al. (2016) (LABS-2) [65]	Shorter diabetes duration Better pre-operative glycemic control No insulin treatment (AGB > RYGB) Baseline weight (AGB) Preserved insulin secretory function (RYGB)	Baseline BMI (RYGB) Preserved insulin secretory function (AGB)
Madesin et al. (2019) [67]	Younger age Shorter diabetes duration Better pre-operative glycemic control No use of oral antidiabetic agents or insulin treatment on baseline	Sex Charlson comorbidity index History of depression or other psychiatric disorders

BMI: body mass index; T2DM: diabetes mellitus type 2; AGB: adjustable gastric banding; RYGB: Roux-en-Y gastric bypass.

**Table 3:** Reported effects of bariatric surgery on the occurrence of chronic diabetes complications and impact on quality of life.

Study	Follow up	Diabetes Complications	Quality of Life *
Dixon et al. (2002) [44]	1 year		(Beck’s depression inventory, SF-36) Significant improvements in depression Significant improvement on physical health subscales
Schauer et al. (2003) [46]	20 months (median)	50% (self-reported) improvement in diabetic neuropathy symptoms	
Schauer et al. (2000) [105]	16.9 months (mean)		(Moorehead-Ardelt QOL Questionnaire) Quality of life 58% greatly improved, 37% improved, 5% no change
Iaconelli et al. (2011) [49]	10 years	All cases with microalbuminuria at baseline regressed by year 10. 2 new cases. Prevalence increased in the control group; 39.2% vs. 9% new nephropathy cases 4 major CV events vs. none	
Schauer et al. (2012) (STAMPEDE) [51]	5 years	No effect on ophthalmologic outcomes vs. conservative treatment Significantly lower albumin-to-creatinine ratio from baseline in the SG group vs. conservative No change in albuminuria status in any group	(SF-36) Significant improvements in both surgical groups in the physical functioning, general health, and energy–fatigue subscales. Emotional well-being worsened significantly among patients in the gastric bypass group
Mingrone et al. (2012) [73]	5 years	5 major diabetic complications in the medically treated group (including 1 fatal myocardial infarction) vs. one in the RYGB arms	(SF-36) Better scores in physical and emotional aspects of QOL in both surgical arms compared to the medically treated arm
Carlsson et al. (2012) (SOS cohort) [53]	18 years	Reduced rates of chronic diabetes complications in the surgical vs. control groups (HRs 0.44 and 0.65 for incident microvascular and macrovascular complications, respectively)	
Karlsson et al. (2007) (SOS cohort) [104]	10 years		(SOS quality of life survey) at 0.5, 1, 2, 3, 4, 6, 8 and 10 years. Substantial early gain trends in QOL that parallel weight loss. Net gains at 10 years in all QOL domains. Greater improvements in social interaction in surgical than conventional arm at 10 years. Better overall mood scores in surgical group up to 2 years post op. Significantly better depression outcomes in surgical group vs. conventional at 10 years. Non-significant improvement in anxiety scores by year 10.
Adams et al. (2012) [54]	6 years		(SF-36) Marked improvement in physical QOL components compared to controls. No significant changes in mental QOL components
Halperin et al. (2014) [61]	1 year		(SF-36, PAID, EQ-5D, IWQOL) No significant differences between RYGB and intensive lifestyle management in components of SF36, PAID, EQ-5D. Greater improvement in IWQOL in RYGB correlated with BMI change
Risstad et al. (2015) [62]	5 years		(SF-36) Similar improvements for RYGB and BPD in components of the SF36 and Obesity–related Problems Scale
Cummings et al. (2016) (CROSSROADS) [64]	1 year		(EQ-5D) Similarly significant QOL improvements for RYGB and ILMI
Salminen et al. (2018) (SLEEVEPASS) [66]	5 years		(Moorehead-Ardelt QOL questionnaire) Similar improvements regarding QOL in VSG and RYGB
Madesin et al. (2019) [67]	5 years	47% lower risk of microvascular complications in RYBG vs. controls Statistically non-significant 24% reduction in macrovascular complications in RYGB vs. controls	

* Text in parentheses indicate the quality of life assessment instrument used. QOL: quality of life; BMI: body mass index; BPD: biliopancreatic diversion; RYGB: Roux-en-Y gastric bypass; SG: sleeve gastrectomy; SOS: Swedish Obese Subjects; SF-36: Short Form (36) Health Survey; PAID: Problem Areas In Diabetes scale; EQ-5D: EuroQol-5D instrument; IWQOL: Impact of Weight on Quality of Life questionnaire.

## References

[1] Ng M., Fleming T., Robinson M., Thomson B., Graetz N., Margono C., Mullany E.C., Biryukov S., Abbafati C., Abera S.F. (2014). Global, regional, and national prevalence of overweight and obesity in children and adults during 1980–2013: A systematic analysis for the Global Burden of Disease Study 2013. Lancet.

[2] Field A.E., Coakley E.H., Must A., Spadano J.L., Laird N., Dietz W.H., Rimm E., Colditz G.A. (2001). Impact of overweight on the risk of developing common chronic diseases during a 10-year period. Arch. Intern. Med..

[3] Verma S., Hussain M.E. (2017). Obesity and diabetes: An update. Diabetes Metab. Syndr..

[4] Barnes A.S. (2011). The epidemic of obesity and diabetes: Trends and treatments. Tex. Heart Inst. J..

[5] Hu F.B. (2011). Globalization of diabetes: The role of diet, lifestyle, and genes. Diabetes Care.

[6] Unnikrishnan R., Pradeepa R., Joshi S.R., Mohan V. (2017). Type 2 Diabetes: Demystifying the Global Epidemic. Diabetes.

[7] Nagaya T., Yoshida H., Takahashi H., Kawai M. (2005). Increases in body mass index, even within non-obese levels, raise the risk for Type 2 diabetes mellitus: A follow-up study in a Japanese population. Diabet. Med..

[8] Farag Y.M., Gaballa M.R. (2011). Diabesity: An overview of a rising epidemic. Nephrol. Dial. Transplant..

[9] American Diabetes Association (2019). 8. Obesity Management for the Treatment of Type 2 Diabetes: Standards of Medical Care in Diabetes-2019. Diabetes Care.

[10] Hall K.D., Kahan S. (2018). Maintenance of Lost Weight and Long-Term Management of Obesity. Med. Clin. North Am..

[11] Rubino F., Nathan D.M., Eckel R.H., Schauer P.R., Alberti K.G., Zimmet P.Z., Del Prato S., Ji L., Sadikot S.M., Herman W.H. (2016). Metabolic Surgery in the Treatment Algorithm for Type 2 Diabetes: A Joint Statement by International Diabetes Organizations. Diabetes Care.

[12] Tadross J.A., le Roux C.W. (2009). The mechanisms of weight loss after bariatric surgery. Int. J. Obes..

[13] Ponce J., DeMaria E.J., Nguyen N.T., Hutter M., Sudan R., Morton J.M. (2016). American Society for Metabolic and Bariatric Surgery estimation of bariatric surgery procedures in 2015 and surgeon workforce in the United States. Surg. Obes. Relat. Dis..

[14] Scopinaro N., Gianetta E., Civalleri D., Bonalumi U., Bachi V. (1979). Bilio-pancreatic bypass for obesity: II. Initial experience in man. Br. J. Surg..

[15] Moshiri M., Osman S., Robinson T.J., Khandelwal S., Bhargava P., Rohrmann C.A. (2013). Evolution of bariatric surgery: A historical perspective. AJR Am. J. Roentgenol..

[16] Quevedo M.D.P., Palermo M., Serra E., Ackermann M.A. (2017). Metabolic surgery: Gastric bypass for the treatment of type 2 diabetes mellitus. Transl. Gastroenterol. Hepatol..

[17] Lean M.E., Leslie W.S., Barnes A.C., Brosnahan N., Thom G., McCombie L., Peters C., Zhyzhneuskaya S., Al-Mrabeh A., Hollingsworth K.G. (2018). Primary care-led weight management for remission of type 2 diabetes (DiRECT): An open-label, cluster-randomised trial. Lancet.

[18] Lean M.E.J., Leslie W.S., Barnes A.C., Brosnahan N., Thom G., McCombie L., Peters C., Zhyzhneuskaya S., Al-Mrabeh A., Hollingsworth K.G. (2019). Durability of a primary care-led weight-management intervention for remission of type 2 diabetes: 2-year results of the DiRECT open-label, cluster-randomised trial. Lancet Diabetes Endocrinol..

[19] Miras A.D., le Roux C.W. (2013). Mechanisms underlying weight loss after bariatric surgery. Nat. Rev. Gastroenterol. Hepatol..

[20] Rabl C., Rao M.N., Schwarz J.M., Mulligan K., Campos G.M. (2014). Thermogenic changes after gastric bypass, adjustable gastric banding or diet alone. Surgery.

[21] Faria S.L., Faria O.P., Cardeal Mde A., Ito M.K., Buffington C. (2014). Diet-induced thermogenesis and respiratory quotient after Roux-en-Y gastric bypass surgery: A prospective study. Surg. Obes. Relat. Dis..

[22] Werling M., Fandriks L., Olbers T., Bueter M., Sjostrom L., Lonroth H., Wallenius V., Stenlof K., le Roux C.W. (2015). Roux-en-Y Gastric Bypass Surgery Increases Respiratory Quotient and Energy Expenditure during Food Intake. PLoS ONE.

[23] Lim E.L., Hollingsworth K.G., Aribisala B.S., Chen M.J., Mathers J.C., Taylor R. (2011). Reversal of type 2 diabetes: Normalisation of beta cell function in association with decreased pancreas and liver triacylglycerol. Diabetologia.

[24] Jackness C., Karmally W., Febres G., Conwell I.M., Ahmed L., Bessler M., McMahon D.J., Korner J. (2013). Very low-calorie diet mimics the early beneficial effect of Roux-en-Y gastric bypass on insulin sensitivity and beta-cell Function in type 2 diabetic patients. Diabetes.

[25] Basso N., Capoccia D., Rizzello M., Abbatini F., Mariani P., Maglio C., Coccia F., Borgonuovo G., De Luca M.L., Asprino R. (2011). First-phase insulin secretion, insulin sensitivity, ghrelin, GLP-1, and PYY changes 72 h after sleeve gastrectomy in obese diabetic patients: The gastric hypothesis. Surg. Endosc..

[26] Laferrere B., Teixeira J., McGinty J., Tran H., Egger J.R., Colarusso A., Kovack B., Bawa B., Koshy N., Lee H. (2008). Effect of weight loss by gastric bypass surgery versus hypocaloric diet on glucose and incretin levels in patients with type 2 diabetes. J. Clin. Endocrinol. Metab..

[27] Shah M., Law J.H., Micheletto F., Sathananthan M., Dalla Man C., Cobelli C., Rizza R.A., Camilleri M., Zinsmeister A.R., Vella A. (2014). Contribution of endogenous glucagon-like peptide 1 to glucose metabolism after Roux-en-Y gastric bypass. Diabetes.

[28] Sam A.H., Gunner D.J., King A., Persaud S.J., Brooks L., Hostomska K., Ford H.E., Liu B., Ghatei M.A., Bloom S.R. (2012). Selective ablation of peptide YY cells in adult mice reveals their role in beta cell survival. Gastroenterology.

[29] Liu L., Wang Y., Wang L., Lin Y., Liu X., Liu X., Liu L. (2013). Exendin-4 protects murine pancreatic beta-cells from free fatty acid-induced apoptosis through PI-3K signaling. Endocr. Res..

[30] Cornu M., Thorens B. (2009). GLP-1 protects beta-cells against apoptosis by enhancing the activity of an IGF-2/IGF1-receptor autocrine loop. Islets.

[31] Drucker D.J. (2003). Glucagon-like peptide-1 and the islet beta-cell: Augmentation of cell proliferation and inhibition of apoptosis. Endocrinology.

[32] Ramracheya R.D., McCulloch L.J., Clark A., Wiggins D., Johannessen H., Olsen M.K., Cai X., Zhao C.M., Chen D., Rorsman P. (2016). PYY-Dependent Restoration of Impaired Insulin and Glucagon Secretion in Type 2 Diabetes following Roux-En-Y Gastric Bypass Surgery. Cell Rep..

[33] Lindqvist A., Spegel P., Ekelund M., Garcia Vaz E., Pierzynowski S., Gomez M.F., Mulder H., Hedenbro J., Groop L., Wierup N. (2014). Gastric bypass improves beta-cell function and increases beta-cell mass in a porcine model. Diabetes.

[34] Perakakis N., Kokkinos A., Peradze N., Tentolouris N., Ghaly W., Tsilingiris D., Alexandrou A., Mantzoros C.S. (2019). Follistatins in glucose regulation in healthy and obese individuals. Diabetes Obes. Metab..

[35] Chondronikola M., Harris L.L., Klein S. (2016). Bariatric surgery and type 2 diabetes: Are there weight loss-independent therapeutic effects of upper gastrointestinal bypass?. J. Intern. Med..

[36] American Diabetes Association (2019). 2. Classification and Diagnosis of Diabetes: Standards of Medical Care in Diabetes-2019. Diabetes Care.

[37] Turner R.C., Cull C.A., Frighi V., Holman R.R. (1999). Glycemic control with diet, sulfonylurea, metformin, or insulin in patients with type 2 diabetes mellitus: Progressive requirement for multiple therapies (UKPDS 49). UK Prospective Diabetes Study (UKPDS) Group. JAMA.

[38] Buse J.B., Caprio S., Cefalu W.T., Ceriello A., Del Prato S., Inzucchi S.E., McLaughlin S., Phillips G.L., Robertson R.P., Rubino F. (2009). How do we define cure of diabetes?. Diabetes Care.

[39] Bruns D.E., Knowler W.C. (2009). Stabilization of glucose in blood samples: Why it matters. Clin. Chem..

[40] Weykamp C. (2013). HbA1c: A review of analytical and clinical aspects. Ann. Lab. Med..

[41] Kwon Y., Kim H.J., Lo Menzo E., Park S., Szomstein S., Rosenthal R.J. (2014). Anemia, iron and vitamin B12 deficiencies after sleeve gastrectomy compared to Roux-en-Y gastric bypass: A meta-analysis. Surg. Obes. Relat. Dis..

[42] Pories W.J., Swanson M.S., MacDonald K.G., Long S.B., Morris P.G., Brown B.M., Barakat H.A., deRamon R.A., Israel G., Dolezal J.M. (1995). Who would have thought it? An operation proves to be the most effective therapy for adult-onset diabetes mellitus. Ann. Surg..

[43] Wittgrove A.C., Clark G.W. (2000). Laparoscopic gastric bypass, Roux-en-Y- 500 patients: Technique and results, with 3-60 month follow-up. Obes. Surg..

[44] Dixon J.B., O’Brien P.E. (2002). Health outcomes of severely obese type 2 diabetic subjects 1 year after laparoscopic adjustable gastric banding. Diabetes Care.

[45] Sugerman H.J., Wolfe L.G., Sica D.A., Clore J.N. (2003). Diabetes and hypertension in severe obesity and effects of gastric bypass-induced weight loss. Ann. Surg..

[46] Schauer P.R., Burguera B., Ikramuddin S., Cottam D., Gourash W., Hamad G., Eid G.M., Mattar S., Ramanathan R., Barinas-Mitchel E. (2003). Effect of laparoscopic Roux-en Y gastric bypass on type 2 diabetes mellitus. Ann. Surg..

[47] Scopinaro N., Marinari G.M., Camerini G.B., Papadia F.S., Adami G.F. (2005). Specific effects of biliopancreatic diversion on the major components of metabolic syndrome: A long-term follow-up study. Diabetes Care.

[48] Dixon J.B., O’Brien P.E., Playfair J., Chapman L., Schachter L.M., Skinner S., Proietto J., Bailey M., Anderson M. (2008). Adjustable gastric banding and conventional therapy for type 2 diabetes: A randomized controlled trial. JAMA.

[49] Iaconelli A., Panunzi S., De Gaetano A., Manco M., Guidone C., Leccesi L., Gniuli D., Nanni G., Castagneto M., Ghirlanda G. (2011). Effects of bilio-pancreatic diversion on diabetic complications: A 10-year follow-up. Diabetes Care.

[50] Kehagias I., Karamanakos S.N., Argentou M., Kalfarentzos F. (2011). Randomized clinical trial of laparoscopic Roux-en-Y gastric bypass versus laparoscopic sleeve gastrectomy for the management of patients with BMI < 50 kg/m^2^. Obes. Surg..

[51] Schauer P.R., Kashyap S.R., Wolski K., Brethauer S.A., Kirwan J.P., Pothier C.E., Thomas S., Abood B., Nissen S.E., Bhatt D.L. (2012). Bariatric surgery versus intensive medical therapy in obese patients with diabetes. N. Engl. J. Med..

[52] Mingrone G., Panunzi S., De Gaetano A., Guidone C., Iaconelli A., Leccesi L., Nanni G., Pomp A., Castagneto M., Ghirlanda G. (2012). Bariatric surgery versus conventional medical therapy for type 2 diabetes. N. Engl. J. Med..

[53] Carlsson L.M., Peltonen M., Ahlin S., Anveden A., Bouchard C., Carlsson B., Jacobson P., Lonroth H., Maglio C., Naslund I. (2012). Bariatric surgery and prevention of type 2 diabetes in Swedish obese subjects. N. Engl. J. Med..

[54] Adams T.D., Davidson L.E., Litwin S.E., Kolotkin R.L., LaMonte M.J., Pendleton R.C., Strong M.B., Vinik R., Wanner N.A., Hopkins P.N. (2012). Health benefits of gastric bypass surgery after 6 years. JAMA.

[55] Gregg E.W., Chen H., Wagenknecht L.E., Clark J.M., Delahanty L.M., Bantle J., Pownall H.J., Johnson K.C., Safford M.M., Kitabchi A.E. (2012). Association of an intensive lifestyle intervention with remission of type 2 diabetes. JAMA.

[56] Arterburn D.E., Bogart A., Sherwood N.E., Sidney S., Coleman K.J., Haneuse S., O’Connor P.J., Theis M.K., Campos G.M., McCulloch D. (2013). A multisite study of long-term remission and relapse of type 2 diabetes mellitus following gastric bypass. Obes. Surg..

[57] Liang Z., Wu Q., Chen B., Yu P., Zhao H., Ouyang X. (2013). Effect of laparoscopic Roux-en-Y gastric bypass surgery on type 2 diabetes mellitus with hypertension: A randomized controlled trial. Diabetes Res. Clin. Pract..

[58] Arterburn D., Bogart A., Coleman K.J., Haneuse S., Selby J.V., Sherwood N.E., Sidney S., Theis M.K., Campos G.M., McCulloch D. (2013). Comparative effectiveness of bariatric surgery vs. nonsurgical treatment of type 2 diabetes among severely obese adults. Obes. Res. Clin. Pract..

[59] Wentworth J.M., Playfair J., Laurie C., Ritchie M.E., Brown W.A., Burton P., Shaw J.E., O’Brien P.E. (2014). Multidisciplinary diabetes care with and without bariatric surgery in overweight people: A randomised controlled trial. Lancet Diabetes Endocrinol..

[60] Courcoulas A.P., Goodpaster B.H., Eagleton J.K., Belle S.H., Kalarchian M.A., Lang W., Toledo F.G., Jakicic J.M. (2014). Surgical vs medical treatments for type 2 diabetes mellitus: A randomized clinical trial. JAMA Surg..

[61] Halperin F., Ding S.A., Simonson D.C., Panosian J., Goebel-Fabbri A., Wewalka M., Hamdy O., Abrahamson M., Clancy K., Foster K. (2014). Roux-en-Y gastric bypass surgery or lifestyle with intensive medical management in patients with type 2 diabetes: Feasibility and 1-year results of a randomized clinical trial. JAMA Surg..

[62] Risstad H., Sovik T.T., Engstrom M., Aasheim E.T., Fagerland M.W., Olsen M.F., Kristinsson J.A., le Roux C.W., Bohmer T., Birkeland K.I. (2015). Five-year outcomes after laparoscopic gastric bypass and laparoscopic duodenal switch in patients with body mass index of 50 to 60: A randomized clinical trial. JAMA Surg..

[63] Yska J.P., van Roon E.N., de Boer A., Leufkens H.G., Wilffert B., de Heide L.J., de Vries F., Lalmohamed A. (2015). Remission of Type 2 Diabetes Mellitus in Patients After Different Types of Bariatric Surgery: A Population-Based Cohort Study in the United Kingdom. JAMA Surg..

[64] Cummings D.E., Arterburn D.E., Westbrook E.O., Kuzma J.N., Stewart S.D., Chan C.P., Bock S.N., Landers J.T., Kratz M., Foster-Schubert K.E. (2016). Gastric bypass surgery vs intensive lifestyle and medical intervention for type 2 diabetes: The CROSSROADS randomised controlled trial. Diabetologia.

[65] Purnell J.Q., Selzer F., Wahed A.S., Pender J., Pories W., Pomp A., Dakin G., Mitchell J., Garcia L., Staten M.A. (2016). Type 2 Diabetes Remission Rates After Laparoscopic Gastric Bypass and Gastric Banding: Results of the Longitudinal Assessment of Bariatric Surgery Study. Diabetes Care.

[66] Salminen P., Helmio M., Ovaska J., Juuti A., Leivonen M., Peromaa-Haavisto P., Hurme S., Soinio M., Nuutila P., Victorzon M. (2018). Effect of Laparoscopic Sleeve Gastrectomy vs Laparoscopic Roux-en-Y Gastric Bypass on Weight Loss at 5 Years Among Patients With Morbid Obesity: The SLEEVEPASS Randomized Clinical Trial. JAMA.

[67] Madsen L.R., Baggesen L.M., Richelsen B., Thomsen R.W. (2019). Effect of Roux-en-Y gastric bypass surgery on diabetes remission and complications in individuals with type 2 diabetes: A Danish population-based matched cohort study. Diabetologia.

[68] Sjostrom L., Lindroos A.K., Peltonen M., Torgerson J., Bouchard C., Carlsson B., Dahlgren S., Larsson B., Narbro K., Sjostrom C.D. (2004). Lifestyle, diabetes, and cardiovascular risk factors 10 years after bariatric surgery. N. Engl. J. Med..

[69] Sjostrom L., Peltonen M., Jacobson P., Ahlin S., Andersson-Assarsson J., Anveden A., Bouchard C., Carlsson B., Karason K., Lonroth H. (2014). Association of bariatric surgery with long-term remission of type 2 diabetes and with microvascular and macrovascular complications. JAMA.

[70] Adams T.D., Gress R.E., Smith S.C., Halverson R.C., Simper S.C., Rosamond W.D., Lamonte M.J., Stroup A.M., Hunt S.C. (2007). Long-term mortality after gastric bypass surgery. N. Engl. J. Med..

[71] Schauer P.R., Bhatt D.L., Kirwan J.P., Wolski K., Brethauer S.A., Navaneethan S.D., Aminian A., Pothier C.E., Kim E.S., Nissen S.E. (2014). Bariatric surgery versus intensive medical therapy for diabetes—3-year outcomes. N. Engl. J. Med..

[72] Schauer P.R., Bhatt D.L., Kirwan J.P., Wolski K., Aminian A., Brethauer S.A., Navaneethan S.D., Singh R.P., Pothier C.E., Nissen S.E. (2017). Bariatric Surgery versus Intensive Medical Therapy for Diabetes—5-Year Outcomes. N. Engl. J. Med..

[73] Mingrone G., Panunzi S., De Gaetano A., Guidone C., Iaconelli A., Nanni G., Castagneto M., Bornstein S., Rubino F. (2015). Bariatric-metabolic surgery versus conventional medical treatment in obese patients with type 2 diabetes: 5 year follow-up of an open-label, single-centre, randomised controlled trial. Lancet.

[74] Courcoulas A.P., Belle S.H., Neiberg R.H., Pierson S.K., Eagleton J.K., Kalarchian M.A., DeLany J.P., Lang W., Jakicic J.M. (2015). Three-Year Outcomes of Bariatric Surgery vs Lifestyle Intervention for Type 2 Diabetes Mellitus Treatment: A Randomized Clinical Trial. JAMA Surg..

[75] Panunzi S., Carlsson L., De Gaetano A., Peltonen M., Rice T., Sjostrom L., Mingrone G., Dixon J.B. (2016). Determinants of Diabetes Remission and Glycemic Control After Bariatric Surgery. Diabetes Care.

[76] Debedat J., Sokolovska N., Coupaye M., Panunzi S., Chakaroun R., Genser L., de Turenne G., Bouillot J.L., Poitou C., Oppert J.M. (2018). Long-term Relapse of Type 2 Diabetes After Roux-en-Y Gastric Bypass: Prediction and Clinical Relevance. Diabetes Care.

[77] Blackstone R., Bunt J.C., Cortes M.C., Sugerman H.J. (2012). Type 2 diabetes after gastric bypass: Remission in five models using HbA1c, fasting blood glucose, and medication status. Surg. Obes. Relat. Dis..

[78] Chikunguwo S.M., Wolfe L.G., Dodson P., Meador J.G., Baugh N., Clore J.N., Kellum J.M., Maher J.W. (2010). Analysis of factors associated with durable remission of diabetes after Roux-en-Y gastric bypass. Surg. Obes. Relat. Dis..

[79] Cotillard A., Poitou C., Duchateau-Nguyen G., Aron-Wisnewsky J., Bouillot J.L., Schindler T., Clement K. (2015). Type 2 Diabetes Remission After Gastric Bypass: What Is the Best Prediction Tool for Clinicians?. Obes. Surg..

[80] Aminian A., Brethauer S.A., Kashyap S.R., Kirwan J.P., Schauer P.R. (2014). DiaRem score: External validation. Lancet Diabetes Endocrinol..

[81] Honarmand K., Chetty K., Vanniyasingam T., Anvari M., Chetty V.T. (2017). Type 2 diabetes remission rates 1-year post-Roux-en-Y gastric bypass and validation of the DiaRem score: The Ontario Bariatric Network experience. Clin. Obes..

[82] Sampaio-Neto J., Nassif L.S., Branco-Filho A.J., Bolfarini L.A., Loro L.S., de Souza M.P., Bianco T. (2015). External Validation of the Diarem Score as Remission Predictor of Diabetes Mellitus Type 2 in Obese Patients Undergoing Roux-En-Y Gastric Bypass. Arq. Bras. Cir. Dig..

[83] Pucci A., Tymoszuk U., Cheung W.H., Makaronidis J.M., Scholes S., Tharakan G., Elkalaawy M., Guimaraes M., Nora M., Hashemi M. (2018). Type 2 diabetes remission 2 years post Roux-en-Y gastric bypass and sleeve gastrectomy: The role of the weight loss and comparison of DiaRem and DiaBetter scores. Diabet. Med..

[84] Craig Wood G., Horwitz D., Still C.D., Mirshahi T., Benotti P., Parikh M., Hirsch A.G. (2018). Performance of the DiaRem Score for Predicting Diabetes Remission in Two Health Systems Following Bariatric Surgery Procedures in Hispanic and non-Hispanic White Patients. Obes. Surg..

[85] Aron-Wisnewsky J., Sokolovska N., Liu Y., Comaneshter D.S., Vinker S., Pecht T., Poitou C., Oppert J.M., Bouillot J.L., Genser L. (2017). The advanced-DiaRem score improves prediction of diabetes remission 1 year post-Roux-en-Y gastric bypass. Diabetologia.

[86] Dicker D., Golan R., Aron-Wisnewsky J., Zucker J.D., Sokolowska N., Comaneshter D.S., Yahalom R., Vinker S., Clement K., Rudich A. (2019). Prediction of Long-Term Diabetes Remission After RYGB, Sleeve Gastrectomy, and Adjustable Gastric Banding Using DiaRem and Advanced-DiaRem Scores. Obes. Surg..

[87] Segal-Lieberman G., Segal P., Dicker D. (2016). Revisiting the Role of BMI in the Guidelines for Bariatric Surgery. Diabetes Care.

[88] Panunzi S., De Gaetano A., Carnicelli A., Mingrone G. (2015). Predictors of remission of diabetes mellitus in severely obese individuals undergoing bariatric surgery: Do BMI or procedure choice matter? A meta-analysis. Ann. Surg..

[89] Astiarraga B., Gastaldelli A., Muscelli E., Baldi S., Camastra S., Mari A., Papadia F., Camerini G., Adami G., Scopinaro N. (2013). Biliopancreatic diversion in nonobese patients with type 2 diabetes: Impact and mechanisms. J. Clin. Endocrinol. Metab..

[90] Adami G.F., Camerini G., Papadia F., Catalano M.F., Carlini F., Cordera R., Scopinaro N. (2019). Type 2 Diabetes Remission and Control in Overweight and in Mildly Obese Diabetic Patients at Long-Term Follow-Up After Biliopancreatic Diversion. Obes. Surg..

[91] Vetter M.L., Ritter S., Wadden T.A., Sarwer D.B. (2012). Comparison of Bariatric Surgical Procedures for Diabetes Remission: Efficacy and Mechanisms. Diabetes Spectr..

[92] Buchwald H., Estok R., Fahrbach K., Banel D., Jensen M.D., Pories W.J., Bantle J.P., Sledge I. (2009). Weight and type 2 diabetes after bariatric surgery: Systematic review and meta-analysis. Am. J. Med..

[93] Liaskos C., Koliaki C., Alexiadou K., Argyrakopoulou G., Tentolouris N., Diamantis T., Alexandrou A., Katsilambros N., Kokkinos A. (2018). Roux-en-Y Gastric Bypass Is More Effective than Sleeve Gastrectomy in Improving Postprandial Glycaemia and Lipaemia in Non-diabetic Morbidly Obese Patients: A Short-term Follow-up Analysis. Obes. Surg..

[94] Pournaras D.J., Aasheim E.T., Sovik T.T., Andrews R., Mahon D., Welbourn R., Olbers T., le Roux C.W. (2012). Effect of the definition of type II diabetes remission in the evaluation of bariatric surgery for metabolic disorders. Br. J. Surg..

[95] Mas-Lorenzo A., Benaiges D., Flores-Le-Roux J.A., Pedro-Botet J., Ramon J.M., Parri A., Villatoro M., Chillaron J., Pera M., Grande L. (2014). Impact of different criteria on type 2 diabetes remission rate after bariatric surgery. Obes. Surg..

[96] van de Laar A.W., Acherman Y.I. (2014). Weight loss percentile charts of large representative series: A benchmark defining sufficient weight loss challenging current criteria for success of bariatric surgery. Obes. Surg..

[97] Puzziferri N., Nakonezny P.A., Livingston E.H., Carmody T.J., Provost D.A., Rush A.J. (2008). Variations of weight loss following gastric bypass and gastric band. Ann. Surg..

[98] Admiraal W.M., Celik F., Gerdes V.E., Dallal R.M., Hoekstra J.B., Holleman F. (2012). Ethnic differences in weight loss and diabetes remission after bariatric surgery: A meta-analysis. Diabetes Care.

[99] Morton J.M. (2016). Ethnic Considerations for Metabolic Surgery. Diabetes Care.

[100] Coleman K.J., Haneuse S., Johnson E., Bogart A., Fisher D., O’Connor P.J., Sherwood N.E., Sidney S., Theis M.K., Anau J. (2016). Long-term Microvascular Disease Outcomes in Patients With Type 2 Diabetes After Bariatric Surgery: Evidence for the Legacy Effect of Surgery. Diabetes Care.

[101] Billeter A.T., Eichel S., Scheurlen K.M., Probst P., Kopf S., Muller-Stich B.P. (2019). Meta-analysis of metabolic surgery versus medical treatment for macrovascular complications and mortality in patients with type 2 diabetes. Surg. Obes. Relat. Dis..

[102] Eliasson B., Liakopoulos V., Franzen S., Naslund I., Svensson A.M., Ottosson J., Gudbjornsdottir S. (2015). Cardiovascular disease and mortality in patients with type 2 diabetes after bariatric surgery in Sweden: A nationwide, matched, observational cohort study. Lancet Diabetes Endocrinol..

[103] Liakopoulos V., Franzen S., Svensson A.M., Zethelius B., Ottosson J., Naslund I., Gudbjornsdottir S., Eliasson B. (2017). Changes in risk factors and their contribution to reduction of mortality risk following gastric bypass surgery among obese individuals with type 2 diabetes: A nationwide, matched, observational cohort study. BMJ Open Diabetes Res. Care.

[104] Karlsson J., Taft C., Ryden A., Sjostrom L., Sullivan M. (2007). Ten-year trends in health-related quality of life after surgical and conventional treatment for severe obesity: The SOS intervention study. Int. J. Obes..

[105] Schauer P.R., Ikramuddin S., Gourash W., Ramanathan R., Luketich J. (2000). Outcomes after laparoscopic Roux-en-Y gastric bypass for morbid obesity. Ann. Surg..

[106] Aminian A., Brethauer S.A., Kirwan J.P., Kashyap S.R., Burguera B., Schauer P.R. (2015). How safe is metabolic/diabetes surgery?. Diabetes Obes. Metab..

[107] Longitudinal Assessment of Bariatric Surgery (LABS) Consortium (2009). Perioperative safety in the longitudinal assessment of bariatric surgery. N. Engl. J. Med..

[108] Buchwald H., Avidor Y., Braunwald E., Jensen M.D., Pories W., Fahrbach K., Schoelles K. (2004). Bariatric surgery: A systematic review and meta-analysis. JAMA.

[109] Kim J.H., Wolfe B. (2012). Bariatric/metabolic surgery: Short- and long-term safety. Curr. Atheroscler. Rep..

[110] Lupoli R., Lembo E., Saldalamacchia G., Avola C.K., Angrisani L., Capaldo B. (2017). Bariatric surgery and long-term nutritional issues. World J. Diabetes.

[111] Liakopoulos V., Franzen S., Svensson A.M., Miftaraj M., Ottosson J., Naslund I., Gudbjornsdottir S., Eliasson B. (2019). Pros and cons of gastric bypass surgery in individuals with obesity and type 2 diabetes: Nationwide, matched, observational cohort study. BMJ Open.

[112] Gill H., Kang S., Lee Y., Rosenblat J.D., Brietzke E., Zuckerman H., McIntyre R.S. (2019). The long-term effect of bariatric surgery on depression and anxiety. J. Affect. Disord..

[113] Backman O., Stockeld D., Rasmussen F., Naslund E., Marsk R. (2016). Alcohol and substance abuse, depression and suicide attempts after Roux-en-Y gastric bypass surgery. Br. J. Surg..

[114] Dawes A.J., Maggard-Gibbons M., Maher A.R., Booth M.J., Miake-Lye I., Beroes J.M., Shekelle P.G. (2016). Mental Health Conditions Among Patients Seeking and Undergoing Bariatric Surgery: A Meta-analysis. JAMA.

[115] Lagerros Y.T., Brandt L., Hedberg J., Sundbom M., Boden R. (2017). Suicide, Self-harm, and Depression After Gastric Bypass Surgery: A Nationwide Cohort Study. Ann. Surg..

[116] Lim R.B.C., Zhang M.W.B., Ho R.C.M. (2018). Prevalence of All-Cause Mortality and Suicide among Bariatric Surgery Cohorts: A Meta-Analysis. Int. J. Environ. Res. Public Health.

[117] Neovius M., Bruze G., Jacobson P., Sjoholm K., Johansson K., Granath F., Sundstrom J., Naslund I., Marcus C., Ottosson J. (2018). Risk of suicide and non-fatal self-harm after bariatric surgery: Results from two matched cohort studies. Lancet Diabetes Endocrinol..

[118] Turner P.L., Saager L., Dalton J., Abd-Elsayed A., Roberman D., Melara P., Kurz A., Turan A. (2011). A nomogram for predicting surgical complications in bariatric surgery patients. Obes. Surg..

[119] Sugerman H.J., DeMaria E.J., Kellum J.M., Sugerman E.L., Meador J.G., Wolfe L.G. (2004). Effects of bariatric surgery in older patients. Ann. Surg..

[120] Ramirez A., Roy M., Hidalgo J.E., Szomstein S., Rosenthal R.J. (2012). Outcomes of bariatric surgery in patients >70 years old. Surg. Obes. Relat. Dis..

[121] Marihart C.L., Brunt A.R., Geraci A.A. (2014). Older adults fighting obesity with bariatric surgery: Benefits, side effects, and outcomes. SAGE Open Med..

[122] Karter A.J., Nundy S., Parker M.M., Moffet H.H., Huang E.S. (2014). Incidence of remission in adults with type 2 diabetes: The diabetes & aging study. Diabetes Care.

[123] Keidar A. (2011). Bariatric surgery for type 2 diabetes reversal: The risks. Diabetes Care.

[124] Tang Q., Sun Z., Zhang N., Xu G., Song P., Xu L., Tang W. (2016). Cost-Effectiveness of Bariatric Surgery for Type 2 Diabetes Mellitus: A Randomized Controlled Trial in China. Medicine.

[125] Gulliford M.C., Charlton J., Prevost T., Booth H., Fildes A., Ashworth M., Littlejohns P., Reddy M., Khan O., Rudisill C. (2017). Costs and Outcomes of Increasing Access to Bariatric Surgery: Cohort Study and Cost-Effectiveness Analysis Using Electronic Health Records. Value Health.

[126] Hoerger T.J., Zhang P., Segel J.E., Kahn H.S., Barker L.E., Couper S. (2010). Cost-effectiveness of bariatric surgery for severely obese adults with diabetes. Diabetes Care.

[127] Keating C., Neovius M., Sjoholm K., Peltonen M., Narbro K., Eriksson J.K., Sjostrom L., Carlsson L.M. (2015). Health-care costs over 15 years after bariatric surgery for patients with different baseline glucose status: Results from the Swedish Obese Subjects study. Lancet Diabetes Endocrinol..

[128] Villamizar N., Pryor A.D. (2011). Safety, effectiveness, and cost effectiveness of metabolic surgery in the treatment of type 2 diabetes mellitus. J. Obes..

[129] Miras A.D., Perez-Pevida B., Aldhwayan M., Kamocka A., McGlone E.R., Al-Najim W., Chahal H., Batterham R.L., McGowan B., Khan O. (2019). Adjunctive liraglutide treatment in patients with persistent or recurrent type 2 diabetes after metabolic surgery (GRAVITAS): A randomised, double-blind, placebo-controlled trial. Lancet Diabetes Endocrinol..

